# Novel Treatment of a Vaccinia Virus Infection from an Occupational Needlestick — San Diego, California, 2019

**DOI:** 10.15585/mmwr.mm6842a2

**Published:** 2019-10-25

**Authors:** Erin R. Whitehouse, Agam K. Rao, Yon C. Yu, Patricia A. Yu, Margaret Griffin, Susan Gorman, Kristen A. Angel, Eric C. McDonald, Anna Liza Manlutac, Marie A. de Perio, Andrea M. McCollum, Whitni Davidson, Kimberly Wilkins, Eddy Ortega, Panayampalli S. Satheshkumar, Michael B. Townsend, Marcia Isakari, Brett W. Petersen

**Affiliations:** ^1^Division of High Consequence Pathogens and Pathology, National Center for Emerging and Zoonotic Infectious Diseases, CDC; ^2^Epidemic Intelligence Service, CDC; ^3^Division of Preparedness and Emerging Infections, National Center for Emerging and Zoonotic Infectious Diseases, CDC; ^4^Division of Strategic National Stockpile, Assistant Secretary for Preparedness and Response, U.S. Department of Health and Human Services; ^5^County of San Diego Health and Human Services Agency, San Diego, California; ^6^County of San Diego Public Health Laboratory, San Diego, California; ^7^Division of Field Studies and Engineering, National Institute for Occupational Safety and Health, CDC; ^8^Center of Occupational and Environmental Medicine, University of California San Diego Health, San Diego, California

Vaccinia virus (VACV) is an orthopoxvirus used in smallpox vaccines, as a vector for novel cancer treatments, and for experimental vaccine research (*1*). The Advisory Committee on Immunization Practices (ACIP) recommends smallpox vaccination for laboratory workers who handle replication-competent VACV (*1*). For bioterrorism preparedness, the U.S. government stockpiles tecovirimat, the first Food and Drug Administration–approved antiviral for treatment of smallpox (caused by variola virus and globally eradicated in 1980*^,†^) (*2*). Tecovirimat has activity against other orthopoxviruses and can be administered under a CDC investigational new drug protocol. CDC was notified about an unvaccinated laboratory worker with a needlestick exposure to VACV, who developed a lesion on her left index finger. CDC and partners performed laboratory confirmation, contacted the study sponsor to identify the VACV strain, and provided oversight for the first case of laboratory-acquired VACV treated with tecovirimat plus intravenous vaccinia immunoglobulin (VIGIV). This investigation highlights 1) the misconception among laboratory workers about the virulence of VACV strains; 2) the importance of providing laboratorians with pathogen information and postexposure procedures; and 3) that although tecovirimat can be used to treat VACV infections, its therapeutic benefit remains unclear.

## Case Report

In December 2018, a healthy female laboratorian aged 26 years, after injecting VACV into the tail of a mouse, sustained a needlestick injury to her left index finger from the same needle. The worker immediately rinsed her finger with water for 15 minutes, notified her supervisors, and visited a local emergency department at the recommendation of a supervisor. In September 2018, before starting working with VACV, she received one-on-one counseling with an occupational health physician about the risks associated with working with VACV and was offered vaccination with ACAM2000 (Emergent BioSolutions), but she declined.

Between days 2 and 9 post infection, the patient was evaluated by two community physicians; neither advised her to observe contact precautions to prevent auto-inoculation or secondary transmission. On day 10, she was evaluated at an occupational health clinic with swelling and a single vesicular lesion at the needlestick site. The treating physician contacted CDC and the County of San Diego Health and Human Services Agency, which advised monitoring her for evidence of worsening infection. On day 12, she was treated at a university-based emergency department for fever (100.9°F [38.3°C]), left axillary lymphadenopathy, malaise, pain, and worsening edema of her finger. Health care providers were concerned about progression to compartment syndrome (excessive pressure in an enclosed muscle space, resulting from swelling after an injury), joint infection, or further spread. The specific VACV strain had not been determined, and its effect on the severity of the infection could not be predicted. Because of concern about her worsening symptoms, on day 12, the patient received a single 6,000 IU/kg dose of VIGIV and was started on a 14-day course of twice-daily (600 mg per dose) oral tecovirimat. She also received clindamycin and cephalexin because of concern about possible secondary bacterial infection. Within 48 hours of treatment initiation, the fever and lymphadenopathy resolved, and the local pain and edema decreased. During treatment with tecovirimat and antibiotics, the patient experienced mild side effects (i.e., nausea, loss of appetite, fatigue, myalgia, and pruritus), and pain in her left finger and arm. The occupational health office excluded the patient from laboratory work for approximately 4 months because of local necrosis and the risk for VACV transmission. Areas of necrotic tissue did not fully resolve until day 94 (Figure). Although the patient was not adequately counseled about transmission risk until 10 days after her injury, no secondary transmission or auto-inoculation occurred.

**FIGURE:**
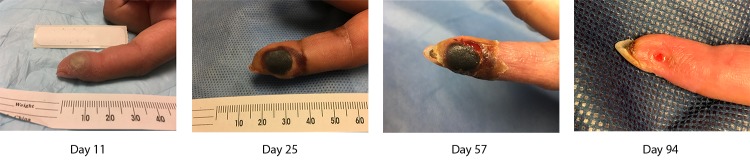
Progression of vaccinia virus infection at 11, 25, 57, and 94 days after an occupational needlestick exposure in December 2018 — San Diego, California, January–April 2019

## Laboratory Analysis

Laboratory verification of VACV infection was performed to rule out other sources of infection, given that the needle pierced a mouse’s tail before piercing the patient’s skin. Swabs collected from the surface of the lesion on days 10 and 12 were submitted to the County of San Diego Public Health Laboratory. Neither sample contained sufficient material for testing. On day 13, the lesion suppurated, and a swab was obtained. Nonvariola orthopoxvirus DNA signatures were amplified using real-time polymerase chain reaction (PCR) testing (Table) (*3*). Additional samples collected from the lesion amplified VACV-specific DNA signatures by real-time PCR. VACV was also obtained by viral culture. Serial serum samples were collected and anti-orthopoxvirus immunoglobulin G and immunoglobulin M antibodies were both present by postexposure day 25 (*4*). The positive immunoglobulin G finding on day 25 and 32 likely reflected administration of VIGIV.

**TABLE:** Laboratory results for vaccinia virus from lesion and serum samples following an occupational needlestick injury to a laboratory work in December 2018 — San Diego, California, January–March 2019

Collection day post infection	PCR result	Viral culture	Serum IgG* (OD-COV)	Serum IgM* (OD-COV)
Day 10	Inconclusive^†,§^	Not done	—	—
Day 12	Inconclusive^†,§^	Not done	Negative (−0.12)	Negative (−0.11)
Day 13	Positive^§^	Not done	—	—
Day 25	Positive^¶^	Positive	Positive (0.897)	Positive (0.096)
Day 28	Positive^¶^	Positive	—	—
Day 32	Positive^¶^	Positive	Positive (0.616)	Positive 0.048)
Day 33	Positive^¶^	Negative	—	—
Day 57	—	—	Positive (0.240)	Equivocal (0.02)
Day 73	Positive^¶^	Not done	—	—

**Abbreviations:** IgG = immunoglobulin G; IgM = immunoglobulin M; OD-COV = optical density cutoff value; PCR = polymerase chain reaction.

* Serum samples were tested by enzyme-linked immunosorbent assay at CDC’s poxvirus laboratory. For IgM, an equivocal OD-COV range exists between 0.00 and 0.04 (https://cvi.asm.org/content/12/7/867).

^†^ Specimen was not positive for human DNA suggesting insufficient sample for testing.

^§^ Nonvariola orthopoxvirus real-time PCR assay.

^¶^ Vaccinia virus–specific real-time PCR assay.

## Occupational Health Investigation

Neither the patient nor the occupational health physician could specify the concentration or strain of VACV preparation used by the patient. Upon inquiry, the study sponsor informed investigators that one of two genetically altered Western Reserve strains could have been involved.^§^ The patient was injecting multiple groups of mice with different strains and did not recall which strain she used when the needlestick injury occurred.

Although the patient had declined vaccination when it was initially offered, during this investigation she reported that she did not appreciate the extent of infection that could occur with VACV when vaccination was first offered. She also cited the challenges of managing the infectious lesion at the vaccination site and potential vaccination adverse events as factors contributing to her initial decision to decline vaccination.

## Discussion

This case was the first use of tecovirimat for a laboratory-acquired VACV infection. Tecovirimat was well tolerated by the patient with mild side effects, even with concurrently administered antibiotics. The patient’s clinical course was similar to previously reported VACV needlestick injuries, but the recovery period was longer (earlier cases resolved within 1–2 months) (*5*–*8*). The VACV strains used by the patient are not known to have heightened virulence, but whether the clinical course would have worsened without VIGIG or tecovirimat is not known. The independent effect of tecovirimat on the clinical course cannot be determined, and whether its use for similar VACV infections would be warranted is not known.

ACIP recommends vaccination for laboratorians who work with replication-competent VACV, unless vaccination is medically contraindicated (*1*); however, laboratories working with VACV set their own policies. ACAM2000 is a live-virus vaccine that produces an infectious vaccination site lesion. The vaccine has very low and known risk of complications for the vaccinee and close contacts (*1*). Appropriate vaccination site care requires careful monitoring of the site and adherence to infection control precautions until the crust separates and a new layer of skin forms.

Counseling before working with VACV needs to include benefits of vaccination, risks of working with VACV in the laboratory, vaccination-associated adverse events, care of the vaccination site, and contraindications to vaccination. Even with counseling, laboratorians might have incomplete understanding of the risks and benefits of vaccination. If the vaccine is medically contraindicated, occupational health providers and laboratorians need to carefully weigh whether continued work with replication-competent VACV is prudent. The complexity of managing a vaccination site might dissuade laboratorians from choosing to receive vaccination. However, accidental inoculations often occur in fingers or eyes, causing infections that present special concern for complications, and clinical management can be difficult (*8*). In addition, laboratory exposures, unlike vaccination, do not have a controlled route of exposure or controlled dose. Previous occupationally acquired VACV infections in unvaccinated workers have required hospitalization, antibiotics for secondary infections, debridement of wounds, and monitoring for functional loss of joints, digits, and vision (*5*,*8*). In one case in which recent vaccination did not fully prevent infection, it did reduce the risk for complications, decrease lesion size, and lead to faster recovery (*7*).

Laboratorians might also underestimate the infection risk from genetically altered, purportedly attenuated VACV strains. Recombinant VACV strains can contain genetic inserts that have unknown or adverse effects on virulence, infectivity, and wound healing (*9*). Most reports of laboratory-acquired VACV infections were caused by thymidine kinase–deletion strains, which are sometimes mistakenly thought to be avirulent or unlikely to cause human infections (*5*,*8*–*10*).

Researchers working with orthopoxviruses need to have information about the virus strains with which they are working and be provided with procedures to follow in the event of an exposure. Information about the specific strain of the VACV can help health care providers and public health officials determine the risks for complications and develop appropriate treatment plans should an infection occur. Laboratories need to implement biosafety policies and procedures and ensure that all personnel are adequately trained and aware of the risks associated with the work they perform (*10*). It is important that biosafety information be posted in the laboratory and adequate disinfectant is available. Providing adequate counseling to laboratorians on vaccination and prompt postexposure assessments requires coordination among laboratories, research universities, and medical providers. In the case reported here, the patient did not initiate contact precautions to prevent auto-inoculation or secondary transmission until treated by an occupational health specialist 10 days after the exposure. Clear postexposure procedures can help ensure prompt care by providers knowledgeable about the treatment of VACV exposures, including implementation of infection control practices to prevent secondary transmission.

**Summary**

**What is already known about this topic?**

Inadvertent exposure to the virus *Vaccinia*, an orthopoxvirus used in biomedical research, can cause considerable injury and time lost from work. Vaccination is recommended for laboratorians using replication-competent vaccinia virus; however, laboratories set their own policies.

**What is added by this report?**

Tecovirimat, a novel antiviral approved for treatment of smallpox, and vaccinia immunoglobulin were used to safely treat an occupational exposure in an unvaccinated laboratorian who was excluded from work for 4 months.

**What are the implications for public health practice?**

Laboratories should ensure that workers are informed of the risks associated with manipulation of vaccinia virus and should counsel workers about the potential benefits of vaccination received according to current guidelines.

## Footnotes

* https://apps.who.int/iris/handle/10665/39253.

§ One strain had a deletion of the thymidine kinase gene; the second had a deletion of the thymidine kinase gene and insertion of mouse hydroxyprostaglandin dehydrogenase 15-(NAD) gene.
